# Development and Validation of a Deep Neural Network for Accurate Identification of Endoscopic Images From Patients With Ulcerative Colitis and Crohn's Disease

**DOI:** 10.3389/fmed.2022.854677

**Published:** 2022-03-18

**Authors:** Guangcong Ruan, Jing Qi, Yi Cheng, Rongbei Liu, Bingqiang Zhang, Min Zhi, Junrong Chen, Fang Xiao, Xiaochun Shen, Ling Fan, Qin Li, Ning Li, Zhujing Qiu, Zhifeng Xiao, Fenghua Xu, Linling Lv, Minjia Chen, Senhong Ying, Lu Chen, Yuting Tian, Guanhu Li, Zhou Zhang, Mi He, Liang Qiao, Zhu Zhang, Dongfeng Chen, Qian Cao, Yongjian Nian, Yanling Wei

**Affiliations:** ^1^Department of Gastroenterology, Daping Hospital, Army Medical University (Third Military Medical University), Chongqing, China; ^2^College of Biomedical Engineering and Imaging Medicine, Army Medical University (Third Military Medical University), Chongqing, China; ^3^Department of Gastroenterology, Sir Run Run Shaw Hospital, Zhejiang University School of Medicine, Hangzhou, China; ^4^Department of Gastroenterology, The First Affiliated Hospital of Chongqing Medical University, Chongqing, China; ^5^Guangdong Provincial Key Laboratory of Colorectal and Pelvic Floor Diseases, Department of Gastroenterology, The Sixth Affiliated Hospital of Sun Yat-sen University, Guangzhou, China; ^6^Department of Gastroenterology, Tongji Hospital of Tongji Medical College, Huazhong University of Science and Technology, Wuhan, China

**Keywords:** inflammatory bowel disease, colonoscopy, deep learning, convolutional neural network, artificial intelligence

## Abstract

**Background and Aim:**

The identification of ulcerative colitis (UC) and Crohn's disease (CD) is a key element interfering with therapeutic response, but it is often difficult for less experienced endoscopists to identify UC and CD. Therefore, we aimed to develop and validate a deep learning diagnostic system trained on a large number of colonoscopy images to distinguish UC and CD.

**Methods:**

This multicenter, diagnostic study was performed in 5 hospitals in China. Normal individuals and active patients with inflammatory bowel disease (IBD) were enrolled. A dataset of 1,772 participants with 49,154 colonoscopy images was obtained between January 2018 and November 2020. We developed a deep learning model based on a deep convolutional neural network (CNN) in the examination. To generalize the applicability of the deep learning model in clinical practice, we compared the deep model with 10 endoscopists and applied it in 3 hospitals across China.

**Results:**

The identification accuracy obtained by the deep model was superior to that of experienced endoscopists per patient (deep model vs. trainee endoscopist, 99.1% vs. 78.0%; deep model vs. competent endoscopist, 99.1% vs. 92.2%, *P* < 0.001) and per lesion (deep model vs. trainee endoscopist, 90.4% vs. 59.7%; deep model vs. competent endoscopist 90.4% vs. 69.9%, *P* < 0.001). In addition, the mean reading time was reduced by the deep model (deep model vs. endoscopists, 6.20 s vs. 2,425.00 s, *P* < 0.001).

**Conclusion:**

We developed a deep model to assist with the clinical diagnosis of IBD. This provides a diagnostic device for medical education and clinicians to improve the efficiency of diagnosis and treatment.

## Introduction

Inflammatory bowel disease (IBD), including ulcerative colitis (UC) and Crohn's disease (CD), is characterized by chronic, relapsing gastrointestinal inflammation. Symptoms include diarrhea, abdominal pain, rectal bleeding, anorexia, and fatigue, which significantly affect the patient's quality of life (1, 2). Although UC and CD share many common symptoms, their differential diagnosis is clinically important due to differences in treatment strategies, predictions of outcomes, comprehensive assessment, and clinical care (3–7). At present, the clinical manifestations of IBD are complicated, and the rate of misdiagnosis is high. Comprehensive analysis and multidisciplinary cooperation are required (8, 9). Precise diagnosis of IBD is the prerequisite and basis of treatment, and it is vital for endoscopists to have a firm grasp of the criteria for specific diagnostic and curative effects (10).

Patients are diagnosed with UC or CD based on endoscopic, histological, clinical, and radiological criteria (11, 12). Colonoscopy plays a crucial role in the diagnosis, treatment, and follow-up monitoring of patients with IBD (13, 14). Digestive endoscopies have been widely used in medical institutions worldwide, and the overall lack of high-level endoscopists and uneven distribution of resources have led primary medical institutions to face certain difficulties in the diagnosis of IBD (5, 15). Unfortunately, endoscopists may misinterpret colonoscopy pictures due to inexperience or subjective unawareness, resulting in missed false diagnoses (false negatives) and delayed treatments (16). It is also possible to interpret nonlesions as lesions (false positives), which not only increases the cost of patients but also increases the risk of disease progression. Therefore, there is a need in gastroenterology healthcare to improve the accuracy of the diagnosis of IBD and to distinguish UC and CD with a sensitive and cost-effective system. This may improve the patient's quality of life and provide endoscopists with medical devices for improving the accuracy and efficiency of diagnosis and treatment (17).

The new era of the healthcare industry has witnessed the explosive development of artificial intelligence (AI), and explorations and applications of deep learning algorithms as a medical assistant tool have demonstrated their ability to optimize the healthcare provided by physicians with a fast and immense processing unit (18). Deep learning-based systems have been most profoundly explored in the gastrointestinal field to reduce the number of missed lesions during colonoscopy. Clinically, the most developed application of endoscopic AI is to assist in differentiating neoplastic and nonneoplastic lesions (19–22). Studies have shown that medical imaging AI-assisted diagnosis can improve the accuracy of diagnosis; reduce missed diagnoses due to workload, fatigue, negligence, and other reasons; and provide a reference for clinicians to make final diagnoses. Although encouraging preliminary results have been published regarding the use of AI in the diagnosis of cancers (21), no research has been reported on the identification of UC and CD. In this study, we aimed to develop and validate a deep learning model to identify IBD types with higher accuracy, sensitivity, and specificity by using endoscopic imaging data from 5 hospitals. In the field of AI, deep learning methods could help primary medical institutions to improve their awareness of disease diagnosis and treatment and appropriately reduce the misdiagnosis rate of digestive endoscopy medical diagnosis. Deep learning methods also have a certain significance for the early diagnosis and treatment of patients. At the same time, the emergence of this model not only optimized medical service but also led to a new generation of medical devices in the field of digestion. Accordingly, this study may provide new perspectives and developments in public health and the medical economy.

## Methods

### Study Design and Participants

**Figure 1:**
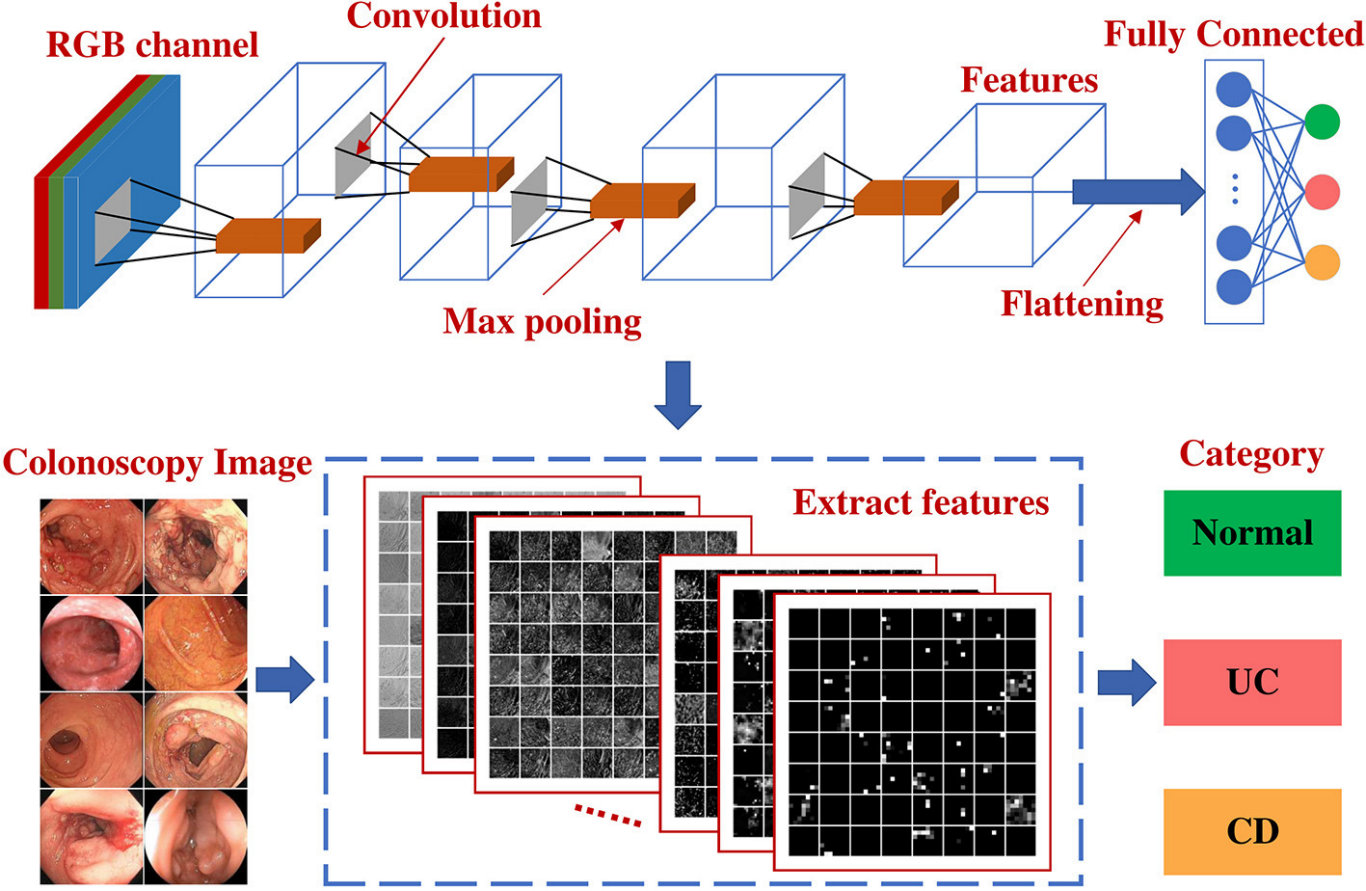
Graphic abstract of the study.

This multicenter, diagnostic retrospective study was performed in 5 hospitals in China. Figure 1 is the graphic abstract of the study. Healthy individuals and active patients with IBD were enrolled, and the disease activity was assessed using the Simple Endoscopic Score for Crohn's Disease (SES-CD) for CD and the Mayo Score for UC. The clinical manifestations of the enrolled patients with IBD showed typical lesions. Patients with IBD unclassified (IBD-U) were excluded. A dataset of 1,576 participants with 47,322 colonoscopy images was obtained between January 2018 and November 2020 from Daping Hospital affiliated with Army Medical University (Chongqing, China) and Sir Run Run Shaw Hospital of Zhejiang University Medical College (Zhejiang, China). Patients met the diagnostic guidelines according to the clinical courses and endoscopic, histopathological, and radiological findings for each disease. We developed a deep learning model based on a deep convolutional neural network (CNN) to identify UC and CD from colonoscopy images. To evaluate the performance of the deep model, we compared our model with 10 endoscopists. To validate the applicability of the deep learning model in clinical practice, from January 2018 to December 2019, 1,832 colonoscopy images from 196 patients (external validation dataset) were also collected from 3 municipalities in China: The First Affiliated Hospital of Chongqing Medical University, Chongqing; Tongji Hospital Affiliated to Huazhong University of Science and Technology, Hubei; and The Sixth Affiliated Hospital of Sun Yat-sen University, Guangzhou. This study was approved by the Ethics Committee of Daping Hospital affiliated with Army Medical University (Third Military Medical University) and was performed according to the Declaration of Helsinki. For patients whose endoscopic images were stored in retrospective databases at each participating hospital, informed consent was exempted by the institutional review boards of the participating hospitals. The study protocol was approved by The Chinese Clinical Trial Registry (http://www.chictr.org/, trial ID: ChiCTR2100043278).

### Colonoscopy and Image Quality Control

All colonoscopy examinations were performed, usually with the patient under sedation, by well-trained endoscopists from the gastroenterology department using high-definition colonoscopes (CV290SL, Olympus Medical Systems, Tokyo, Japan). Colonoscopy records included a written description and a scheme representing the colon where the different lesions (frank erythema, aphtha, superficial and deep ulcerations, pseudopolyp, and stenosis) were displayed for each colonic segment (rectum and sigmoid, descending, transverse, and ascending colon).

### Colonoscopy Patient and Lesion Labeling

Three experienced endoscopists from the Daping Hospital affiliated with Army Medical University were recruited to classify the patients and colonoscopy images into three categories: normal, UC, and CD. Normal endoscopic images from participants had routine physical examination or existing lower gastrointestinal symptoms, such as abdominal pain, diarrhea, constipation, changes in stool habits, and other suspected colorectal diseases, but the results of colonoscopy showed no organic lesions. All three expert endoscopists were trained in IBD diagnostic studies and had more than 10 years of experience in IBD endoscopy. The labeling was based on the patient's entire colonoscopy images, hematoxylin-eosin (H&E)-stained tissue slides, radiological features, and other clinical indicators, as well as the identification from 2 independent endoscopists with experience in IBD diagnosis. During classification, endoscopists were blinded to the study details and patient information. If there was a disagreement between endoscopists during identification, the final label was adjudicated by a third independent endoscopist.

### Deep Model Development and Data Augmentation

Colonoscopy images from the 2 hospitals were randomly assigned to the training and validation datasets for model training and evaluation. We built a classic ResNet50 network (Supplementary Figure 1) and changed the nodes of its output layer to three, representing normal, UC, and CD. Then, we applied the pre-trained weights on the ImageNet dataset to the model so that our network could converge faster on the task of identifying colonoscopy images. The task was based on the PyCharm 2020.1.3 platform, with Python version 3.8.5 and PyTorch framework version 1.7.0. An NVIDIA Quadro GV100 32G graphics card was used to train the network.

Data augmentation can improve the generalization ability of the model and prevent overfitting. In this study, we performed conventional data augmentation operations on the image, such as horizontal flipping, vertical flipping, random cropping, random rotation, brightness adjustment, contrast adjustment, and saturation adjustment. In addition, the CutMix algorithm was also used, which can fuse different sample information and further improve the classification ability of the network. Compared with Mixup and Cutout, it has better performance (23).

### Identification and Model Interpretation

Notably, the deep model outputs the category probability rather than directly outputting the category. Supposing one patient has *k* images, and the probability of belonging to class *j* (*j* = 1, 2, and 3 represent normal, UC, and CD, respectively) of the *i*-th (*i* = 1, 2, …, *k*) image is$p_{i}^{j}$, which can be obtained by our deep model. Let $C_{image}^{m}$ denote the category to which the *m*-th image of this patient belongs, and*C*_*patient*_ denote the category to which this patient belongs. The determination of $C_{image}^{m}$ and *C*_*patient*_ can then be written as follows:


(1) $$\begin{array}{r} C_{image}^{m}=ArgMax\left(p_{m}^{1},p_{m}^{2},p_{m}^{3}\right) \end{array}$$

(2) $$\begin{array}{r} C_{patient}=ArgMax\left(\frac{1}{k}\overset{k}{\underset{i=1}{\sum }}p_{i}^{1},\frac{1}{k}\overset{k}{\underset{i=1}{\sum }}p_{i}^{2},\frac{1}{k}\overset{k}{\underset{i=1}{\sum }}p_{i}^{3}\right) \end{array}$$

where the *ArgMax* operation returns the category index corresponding to the maximum category probability. In clinical diagnosis, the evidence for decision-making is very important. However, it is always difficult to construct deep models to intuitively analyze and identify data. For this reason, we provided a visual interpretation of the deep model, which can output a heatmap to provide lesion localization in the colonoscopy image.

### Test of the Deep Model and Comparison Between Endoscopists and the Model

We first validated the deep model in the identification of IBD lesions in patients using an internal test dataset from 2 hospitals. Moreover, all 218 patients (4,886 images) were read using manual and deep models. The endoscopic images for competent endoscopists and the deep model were screened. All data were randomly assigned to 10 endoscopists: 5 trainee endoscopists with 5 years of experience in endoscopy and 5 competent endoscopists who were attending endoscopists with 5–7 years of experience. All endoscopists were trained in IBD diagnostic studies, completed both clinical and specific endoscopic training, and were not involved in the enrollment and labeling of the patients and images. During the comparison test, all data were randomized and deidentified beforehand. Moreover, we assessed the model using external test datasets from another 3 participating hospitals, each with a small dataset of patients.

### Statistical Analysis

The indices, including diagnostic accuracy, sensitivity, specificity, positive predictive value (PPV), and negative predictive value (NPV) for the identification of lesions and patients, were evaluated using the McNemar test with 95% Wilson confidence intervals (CIs), while the chi-squared test was used to compare the difference in PPV and NPV between the endoscopist and deep model. An independent sample *t*-test was used to compare the identifying time for imaging reading by the deep model and endoscopists. All tests were analyzed using the IBM SPSS Statistics 25.0 software. Receiver operating characteristic (ROC) curves were created by plotting the proportion of true-positive cases (sensitivity) against the proportion of false-positive cases (1-specificity) by varying the predictive probability threshold (24). A *P-*value of <0.05 was considered statistically significant.

## Results

### Patient Enrollment

**Figure 2:**
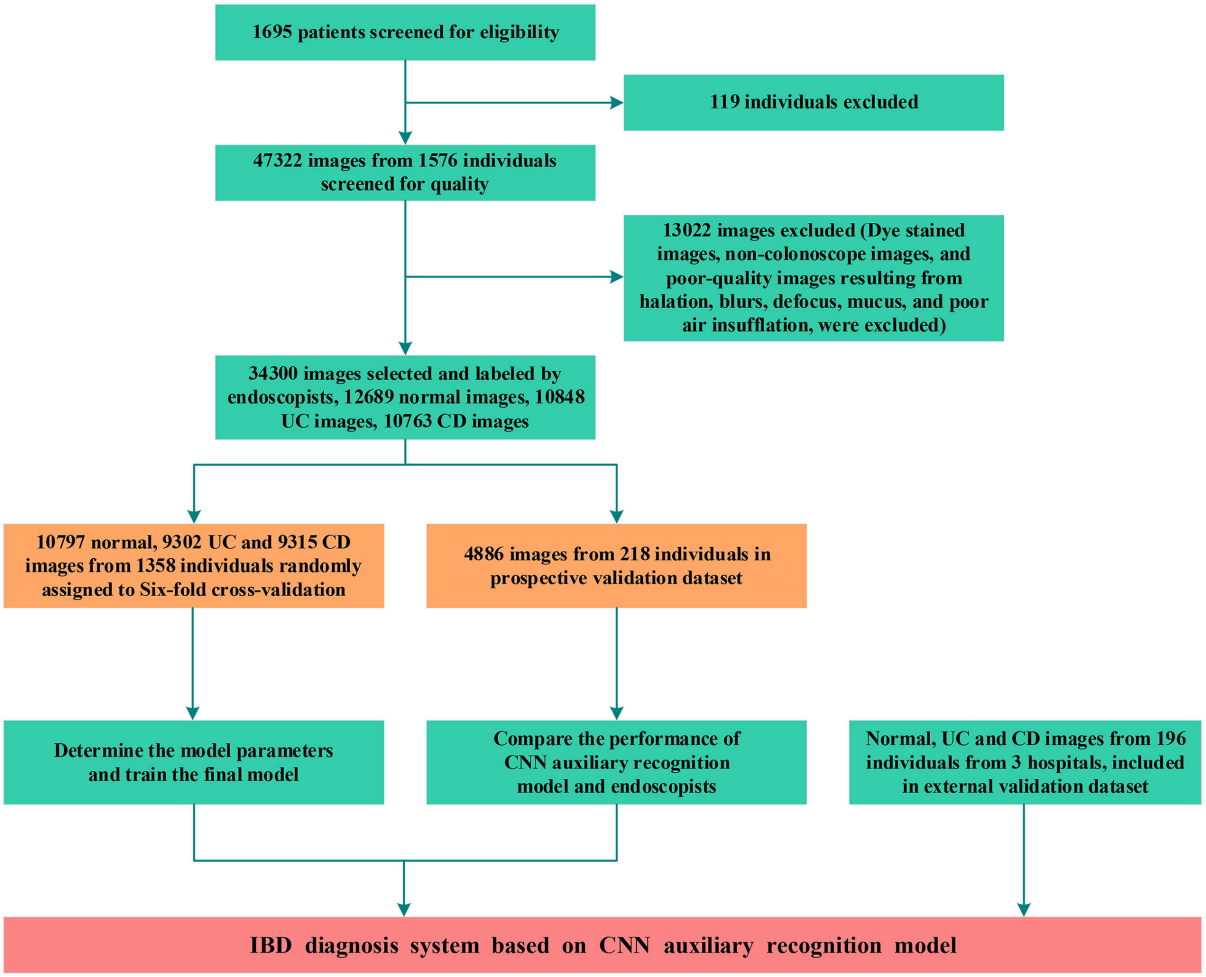
Workflow diagram of the development and evaluation of the deep learning model. UC, ulcerative colitis; CD, Crohn's disease; CNN, convolutional neural network.

Between January 2018 and December 2020, 47,322 images from 1,576 patients were obtained from 2 hospitals. Due to undetermined pathological diagnosis and undiagnosed clinical manifestations, 119 patients were excluded. After quality control evaluation, 13,022 of 47,322 images were discarded. For patients with IBD, only images of lesions were included. For patients without IBD, 12,689 images were enrolled as the normal group. Overall, 29,414 endoscopic images from 1,358 patients were used to build the model, and 4,886 endoscopic images from 218 patients were used for model testing and accuracy comparison by 10 endoscopists. Colonoscopy images from 196 patients in 3 hospitals were used as an external dataset to evaluate the generalization capability of the model (Figure 2). After randomized allocation, the three groups of patient and image characteristics in the training and validation datasets had similar background data regarding age, sex, bowel preparation, clinical severity, and so on (Table 1).

**Table 1:** Patient and image characteristics in the training and validation datasets.

**Patients**	**Normal training datasets** **(*n =* 474)**	**Normal validation datasets** **(*n =* 82)**	**UC training datasets** **(*n =* 440)**	**UC validation datasets** **(*n =* 72)**	**CD training datasets** **(*n =* 444)**	**CD validation datasets** **(*n =* 64)**
Males, *n* Age, y, mean (SD) (%)	238 (42.81%) 42.6 (13.5)		202 (39.45%) 41.8 (14.1)		192 (37.80%) 43.4 (14.7)	
BBPS (mean, median, range)	2.85, 3, (1-3)	2.61, 3, (1-3)	2.50, 3, (1-3)	1.92, 2, (1-3)	2.4, 2, (1-3)	2.0, 2, (1-3)
Disease duration, y, mean (SD)	**–**		2.58 (1.24)	2.38 (1.11)	4.63 (2.35)	4.11 (1.17)
**Disease manifestation**						
Diarrhea, *n* (%)	**–**	**–**	346 (78.64%)	61 (86.11%)	353 (79.50%)	43 (67.19%)
Abdominal pain, *n* (%)	**–**	**–**	322 (73.18%)	56 (77.78%)	262 (66.00%)	49 (76.56%)
CDAI, mean (SD)	**–**	**–**	**–**	**–**	277.56 (104.00)	284.06 (88.01)
SES-CD, mean (SD)	**–**	**–**	**–**	**–**	6.52 (2.52)	5.75 (2.66)
**Mayo endoscopy score**						
1, *n* (%)	**–**	**–**	94 (21.36%)	10 (13.89%)	**–**	**–**
2, *n* (%)	**–**	**–**	48 (10.91%)	11 (15.28%)	**–**	**–**
3, *n* (%)	**–**	**–**	298 (67.73%)	51 (70.83%)	**–**	**–**
Number of images per patient, mean (SD)	23 (2.4)		21 (1.7)		21 (1.2)	

*UC, ulcerative colitis; CD, Crohn's disease, BBPS, Boston bowel preparation score; y, year; SD, standard deviation*.

### Deep Model in the Training and Validation Datasets in Per Patient and Per Lesion Analyses

**Figure 3:**
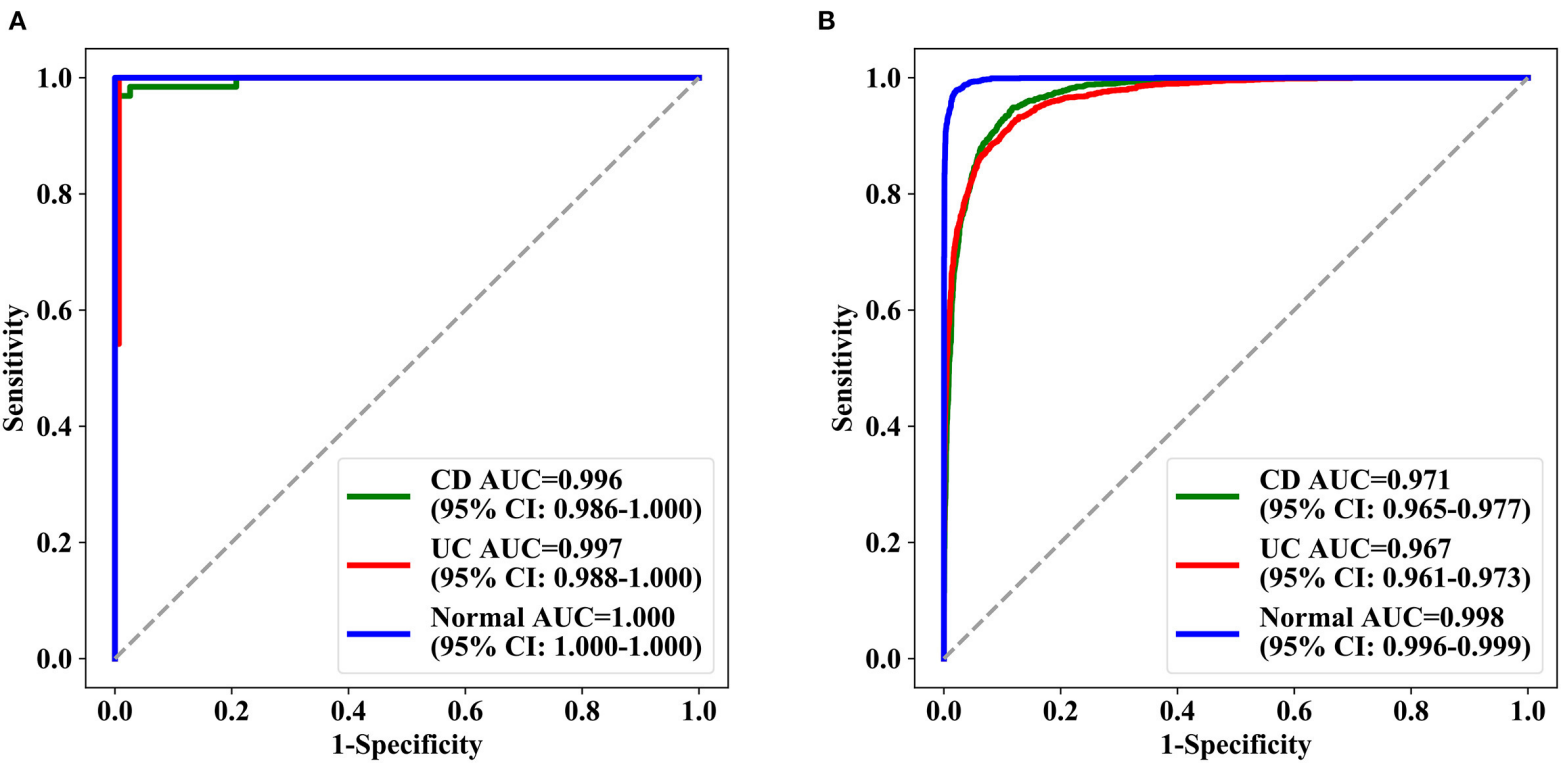
ROC curves achieved by deep model ROC of the three categories of deep learning model in the test set. **(A)** The test results of three groups of patients and **(B)** the test results of the lesions (signal picture). AUC represents the area under the ROC curve, and in parentheses is the 95% confidence interval of AUC. AUC, the area under the receiver operating characteristic curve; ROC, receiver operating characteristic.

A total of 29,414 images from 1,358 patients were used for sixfold cross-validation five times in the training and validation phases, and the diagnostic accuracy per patient and per lesion was 0.962 (95% CI: 0.951–0.971) and 0.916 (95% CI: 0.913–0.920), respectively. In patient analysis, the sensitivity, specificity, PPV, and NPV for the training dataset were over 0.900 (Supplementary Table 1); the AUC values for normal, UC, and CD were 0.996 (95% CI: 0.986–1.000), 0.997 (95% CI: 0.988–1.000), and 1.000 (95% CI: 1.000–1.000), respectively, for the test dataset (Figure 3A). For lesion analyses, the AUC values of normal, UC, and CD were 0.999 (95% CI: 0.999–0.999), 0.974 (95% CI: 0.971–0.976), and 0.977 (95% CI: 0.975–0.980), respectively, for the training dataset, and 0.971 (95% CI: 0.965–0.977), 0.967 (95% CI: 0.961–0.973), and 0.998 (95% CI: 0.996–0.999), respectively, for the test dataset (Figure 3B).

### High Accuracy and Time Efficiency of the Deep Model in the Detection of IBD Compared With Endoscopists

**Figure 4:**
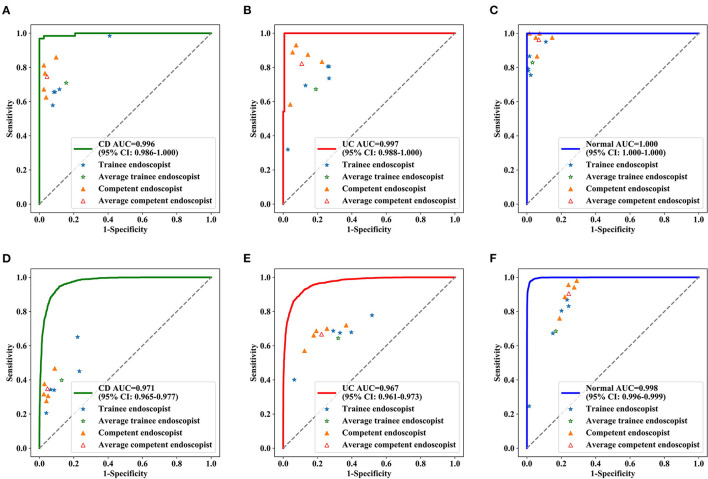
Diagnostic performance of deep model and endoscopists in the test dataset. ROC curve for CD **(A)**, UC **(B)** and normal **(C)** at the patient level, ROC curve for CD **(D)**, UC **(E)**, and normal **(F)** at lesions level. The blue stars indicate the diagnostic sensitivities and specificities of the trainee endoscopists, the green star indicates the pooled sensitivities and specificities of all trainee endoscopists, the yellow triangles indicate the diagnostic sensitivities and specificities of the competent endoscopists, and the red triangle indicates the pooled sensitivities and specificities of all competent endoscopists. AUC, the area under the receiver operating characteristic curve; ROC, receiver operating characteristic.

Identifying IBD per patient and per lesion using a deep model and conventional reading was analyzed according to consensus evaluation. For detecting IBD, the deep model performed significantly better than the trainee (highest accuracy) and competent (highest accuracy) endoscopist in the patient analysis (*P* < 0.001), with an accuracy of 0.991 (95% CI: 0.967–0.997) achieved by the deep model, 0.780 (95% CI: 0.720–0.830) by the trainee endoscopist, and 0.922 (95% CI: 0.879–0.951) by the experienced endoscopist (*P* < 0.001) (Table 2). Our model showed a higher diagnostic performance than conventional reading (Figure 4). The confusion matrix between the deep model and conventional reading is shown in Supplementary Figure 2, and heatmaps demonstrating discriminative image regions are shown in Supplementary Figure 3.

**Table 2:** Comparison of classification performance between deep model and endoscopy doctors (per patient and per lesion).

	**ResNet50**	**Highest trainee endoscopist**	***P-*value**	**Highest competent endoscopist**	***P-*value**
**Per patient analysis**					
Accuracy (95%CI)	0.991 (0.967–0.997)	0.780 (0.720–0.830)	<0.001	0.922 (0.879–0.951)	<0.001
**Normal**					
Sensitivity (95%CI)	1.000 (0.955–1.000)	0.951 (0.881–0.981)	0.125	1.000 (0.955–1.000)	1.000
Specificity (95%CI)	1.000 (0.973–1.000)	0.890 (0.826–0.932)	<0.001	0.985 (0.948–0.996)	0.500
PPV (95%CI)	1.000 (0.955–1.000)	0.839 (0.751–0.900)	<0.001	0.976 (0.917–0.993)	0.497
NPV (95%CI)	1.000 (0.973–1.000)	0.968 (0.921–0.987)	0.051	1.000 (0.972–1.000)	1.000
**UC**					
Sensitivity (95%CI)	1.000 (0.949–1.000)	0.694 (0.580–0.789)	<0.001	0.931 (0.848–0.970)	0.219
Specificity (95%CI)	0.993 (0.962–0.999)	0.870 (0.806–0.915)	<0.001	0.925 (0.870–0.957)	0.002
PPV (95%CI)	0.986 (0.926–0.998)	0.725 (0.610–0.816)	<0.001	0.859 (0.765–0.919)	0.010
NPV (95%CI)	1.000 (0.974–1.000)	0.852 (0.787–0.900)	<0.001	0.964 (0.919–0.985)	0.197
**CD**					
Sensitivity (95%CI)	0.969 (0.893–0.991)	0.656 (0.534–0.761)	<0.001	0.812 (0.700–0.889)	0.001
Specificity (95%CI)	1.000 (0.976–1.000)	0.909 (0.853–0.945)	0.001	0.974 (0.935–0.990)	0.375
PPV (95%CI)	1.000 (0.942–1.000)	0.750 (0.623–0.845)	<0.001	0.929 (0.830–0.972)	0.285
NPV (95%CI)	0.987 (0.954–0.996)	0.864 (0.803–0.909)	<0.001	0.926 (0.875–0.957)	0.006
**Per lesion analysis**					
Accuracy (95%CI)	0.904 (0.895–0.912)	0.597 (0.583–0.610)	<0.001	0.699 (0.686–0.712)	<0.001
**Normal**					
Sensitivity (95%CI)	0.978 (0.971–0.984)	0.831 (0.814–0.848)	<0.001	0.956 (0.946–0.964)	0.090
Specificity (95%CI)	0.979 (0.973–0.984)	0.757 (0.741–0.772)	<0.001	0.758 (0.743–0.773)	<0.001
PPV (95%CI)	0.967 (0.958–0.974)	0.684 (0.665–0.703)	<0.001	0.714 (0.696–0.731)	<0.001
NPV (95%CI)	0.986 (0.981–0.990)	0.877 (0.863–0.889)	<0.001	0.965 (0.956–0.971)	0.001
**UC**					
Sensitivity (95%CI)	0.928 (0.914–0.939)	0.675 (0.652–0.698)	<0.001	0.687 (0.663–0.710)	<0.001
Specificity (95%CI)	0.878 (0.866–0.888)	0.669 (0.653–0.685)	<0.001	0.807 (0.793–0.820)	<0.001
PPV (95%CI)	0.778 (0.759–0.796)	0.486 (0.464–0.507)	<0.001	0.622 (0.599–0.645)	<0.001
NPV (95%CI)	0.963 (0.956–0.969)	0.817 (0.802–0.831)	<0.001	0.848 (0.835–0.860)	<0.001
**CD**					
Sensitivity (95%CI)	0.948 (0.935–0.958)	0.206 (0.186–0.227)	<0.001	0.376 (0.352–0.402)	<0.001
Specificity (95%CI)	0.883 (0.872–0.893)	0.960 (0.953–0.966)	<0.001	0.971 (0.964–0.976)	<0.001
PPV (95%CI)	0.773 (0.753–0.792)	0.683 (0.638–0.725)	<0.001	0.844 (0.814–0.870)	0.901
NPV (95%CI)	0.976 (0.970–0.980)	0.742 (0.729–0.754)	<0.001	0.787 (0.774–0.799)	<0.001

*UC, ulcerative colitis; CD, Crohn's disease; PPV, positive predictive value; NPV, negative predictive value; AUC, area under the receiver operating characteristic curve*.

During conventional reading, an average of 23 images were read from each patient, and the manual reading time was more than 1,000.00 s (trainee and competent endoscopists), whereas the mean reading time by the deep model was 6.00 s (Table 3), which was significantly lower than that of the competent endoscopists and trainee endoscopists (*P* < 0.001).

**Table 3:** Comparison of time consumption for diagnosing the same test dataset (unit: s).

	**Trainee endoscopist (highest)**	**Competent endoscopist (highest)**	**Deep model**	***P*-value (Trainee endoscopist vs. Model)**	***P*-value (Competent endoscopist vs. Model)**
All lesion images	12,862	11,326	6.00	*P < * 0.001	*P < * 0.001
All patients	2,421	2,429	6.20	*P < * 0.001	*P < * 0.001

### Multicenter Verification Achieved by the Deep Model

**Figure 5:**
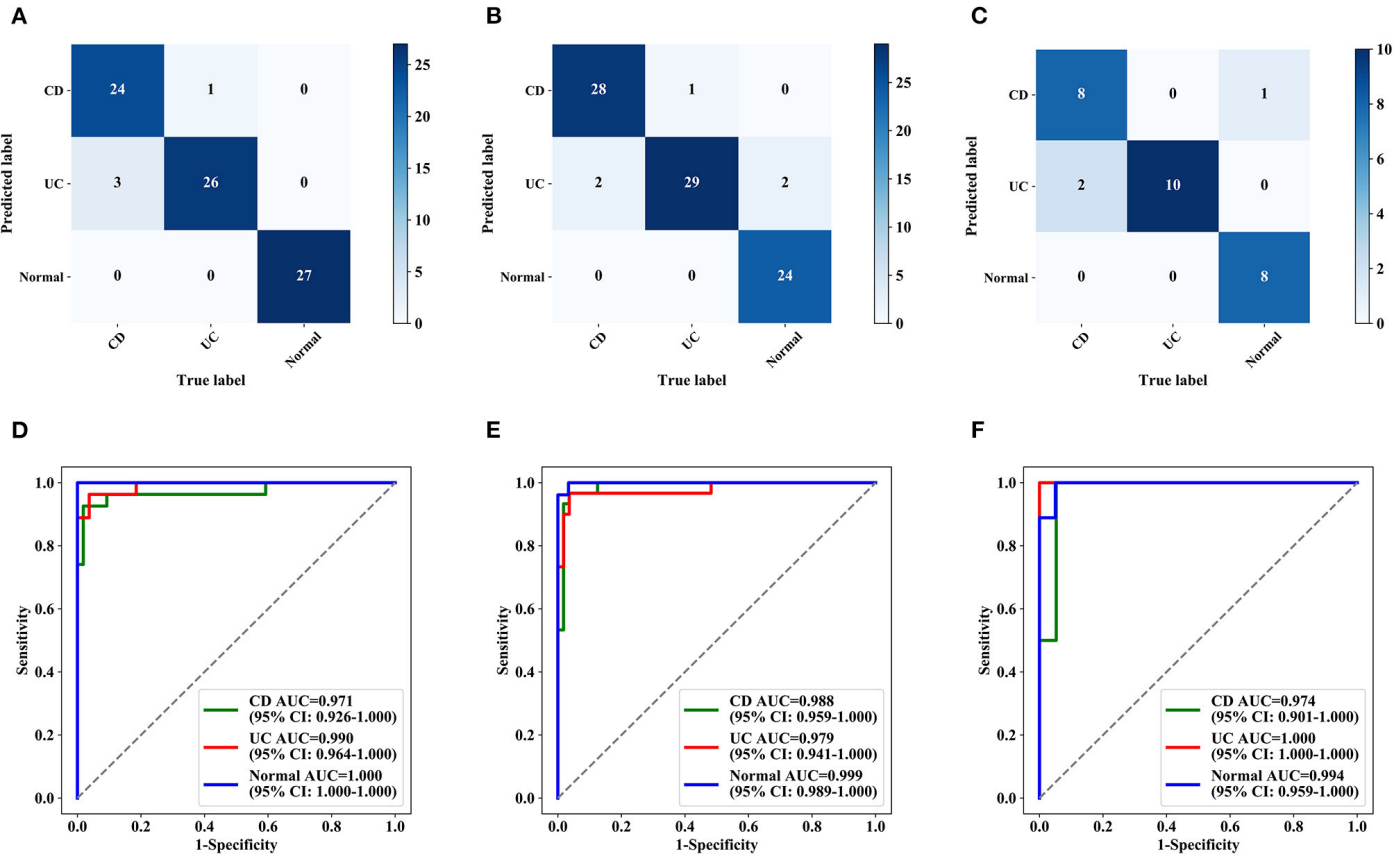
Confusion matrix and ROC curves for multicentre validation (per patient). Confusion matrix **(A–C)** and ROC curves **(D–F)** for three hospitals. **(A,D)** The First Affiliated Hospital of Chongqing Medical University. **(B,E)** The Sixth Affiliated Hospital of Sun Yat-sen University. **(C,F)** Tongji Hospital Affiliated with Huazhong University of Science and Technology. AUC, the area under the receiver operating characteristic curve; ROC, receiver operating characteristic.

The deep model showed high accuracy in the identification of IBD in 3 hospitals during external testing (Supplementary Table 2), with an accuracy of 0.951 (95% CI: 0.880–0.981) for the First Affiliated Hospital of Chongqing Medical University, 0.909 (95% CI: 0.831–0.953) for the Sixth Affiliated Hospital of Sun Yat-sen University, and 0.900 (95% CI: 0.699–0.972) for the Tongji Hospital Affiliated to Huazhong University of Science and Technology. The sensitivity and specificity were higher than 0.80 in all of the test datasets. Multicenter datasets missed diagnoses in 281 images and 12 patients, and the confusion matrix and ROC curve for multicenter validation are shown in Figure 5 and Supplementary Figure 4. The accuracy of the external datasets in the three hospitals was lower than that of the model verification, which seems to be related to the models of endoscopy instruments and equipment setting parameters in each hospital and the operating habits of the endoscopists. The verification set of the top three hospitals has 196 patients with 1,832 colonoscopy images. Although the validation of these three external small-sample datasets was lower than the model validation, it was also similar to the accuracy of human reviewers and deep learning research that has been reported (19, 25). Improving the generalization ability of the model on external data requires more multicenter data for training. This approach is an effective way to further improve model performance, and we plan to pursue this aim in our future work.

### Colonoscopy Diagnosis System

A website with free access to the deep learning colonoscopy diagnosis system was built (http://120.27.8.126/ai/default.aspx). Endoscopists and students can upload colonoscopy images to obtain IBD identification with the possibility of 3 categories (Supplementary Figure 5).

## Discussion

In our study, the deep model proved high accuracy, sensitivity, and specificity in the differential diagnosis of UC and CD based on retrospectively stored images. Moreover, the identification accuracy obtained by the deep model was superior to that of experienced endoscopists per patient and per lesion. In addition, the mean reading time was reduced by the deep model. In comparison, the deep model can be helpful in the rapid identification and retrospective classification of colonoscopy images on a free website. Our research is in an exploratory research stage, and the mature AI medical equipment will require further research. From the findings of our research, we can assist inexperienced physicians in better understanding the endoscopic differences between UC and CD during the endoscopic examination of patients with IBD, improve the accuracy of diagnosis, and assist experienced physicians in making endoscopic diagnoses of UC and CD faster and more efficiently. The findings of this research can also provide a visual clinical teaching and research auxiliary tool. To the best of our knowledge, there is no report on AI-assisted differential diagnosis of IBD based on colonoscopy images. With the development of endoscopic diagnosis, there is a shortage of specialists who can perform high-precision endoscopy. We will examine whether AI with excellent image recognition ability can overcome this problem.

Although some efforts have been made to construct and develop models based on IBD diagnosis, many of them have been stalled due to their retrospective nature, small sample sizes, and single institutional research at similar tier hospitals (26, 27). Tong et al. (22) used random forest (RF) and AI methods to contrast a three-class model that distinguishes UC, CD, and intestinal tuberculosis based on colonoscopy reports. In contrast, we developed and validated a large cohort of more than 34,300 images from different hospitals and exhibited an overall high accuracy of 0.991 (95% CI: 0.967–0.997) for the detection of IBD in three retrospective validation sets, which strongly suggests the generalizability of the system in various scenarios. In this study, our deep model achieved high accuracy, sensitivity, specificity, PPV, and NPV compared with a trainee and experienced endoscopist in the per lesion analysis (*P* < 0.001). Young doctors recognized the performance characteristics of UC and CD lesions. Therefore, the model can serve as a diagnostic tool for teaching or enable clinicians to improve the efficiency of diagnosis and treatment. Moreover, it is our ultimate aim to further provide a real-time auxiliary diagnosis. In clinical diagnosis, it is intuitively difficult for deep models to understand and explain the underlying logic among pictures. The model outputs the probability per image that the image belongs to each category, and the category corresponding to the maximum probability is the final predicted value. However, the endoscopists diagnosed patients based on all the images of this patient, which considered the characteristics of lesions in different parts of the colon. Therefore, we designed the AI system for inexperienced or trainee endoscopists to differentiate and diagnose IBD more efficiently. Through the interpretation of the multicenter verification results, we can see that the characteristics of colonoscopy images are affected by many factors, such as equipment, light, and doctor technology. Nevertheless, a multicenter high-quality study with a larger sample is necessary for further verification.

There are some limitations to our study. First, the differential diagnosis of intestinal inflammatory diseases includes not only UC and CD but also intestinal tuberculosis, Behcet's disease, and amoebiasis. Recently, Kim et al. (23) found that deep-learning models can distinguish between colonoscopy images of intestinal BD, CD, and ITB. Lin et al. (24) presented gastroenterologist-level identification of small-bowel diseases by capsule endoscopy using a deep learning model, and its images were categorized as normal, inflammation, ulcer, polyps, lymphangiectasia, bleeding, vascular disease, protruding lesion, lymphatic follicular hyperplasia, diverticulum, parasite, and others. We designed this study and considered adding colonoscopy images of these diseases, but due to the low incidence, the image collection cycle is long, and we are collecting images to improve the diagnosis system category. Second, Xu et al. (22) showed real-time AI for the detection of upper gastrointestinal cancer by endoscopy. Moreover, our deep model used still images captured by endoscopists after the colonoscopy procedure was completed. Although the results show that the reading time is reduced by this deep model, the pictures have been preselected, which is time-consuming and not included in this analysis. Nevertheless, the endoscopists were able to observe the colon in real-time while withdrawing and had the chance to obtain a much greater impression of the anatomy, size and number of lesions, number of stenoses, and unaffected areas of the colon. This may lead to much higher accuracy for real-time endoscopists. Therefore, the deep model needs to be continuously studied and optimized to improve the diagnostic level of the model. At present, deep models display high accuracy to help less experienced endoscopists grasp the characteristics of IBD and provide endoscopy tools in diagnostic teaching courses. Finally, anatomical location information is important to determine whether CD is present, and we took this limitation while labeling patients and developing models. Since the diagnostic model we build is a static image rather than a video dataset, the location factor is excluded. Unfortunately, the accuracy was reduced when labeling the anatomical location in the rectum and sigmoid, descending, transverse, and ascending colon, and we believe this is why human reviewers are irreplaceable by any machine learning. AI will never be able to think completely like the human brain, and the complexity of the human brain is not comparable to that of the current simple computer. However, AI can assist doctors in dealing with clinical problems more efficiently and nonsubjectively in many clinical fields. In our ongoing research, during real-time deep learning diagnosis, the anatomical location of the colonoscope is manually interpreted and fed back to the model, which might not affect the accuracy of the system. Moreover, it is important to pay attention to the accuracy of deep models at different stages of endoscopic disease severity, which has not been included in this study. This was also a limitation of this study. In the following research, we are collecting and sorting out the data related to the Mayo score of UC and the SES-CD score of CD. We will see data on the accuracy of the system in different stages of endoscopic disease severity. The Mayo score and SES-CD score of UC and CD may have an impact on the classification accuracy.

This study developed an AI system based on colonoscopy images from multiple centers to achieve a differential diagnosis of IBD and the accuracy of auxiliary diagnosis of IBD. Given its high accuracy, fidelity, and stability, the current deep model is potentially applicable in current clinical practice to help endoscopists. This may lay a foundation for AI in IBD endoscopic diagnosis research and provide a new challenge and perspective. Nevertheless, a multicenter high-quality study with a larger sample is necessary for further verification.

## Data Availability Statement

The raw data supporting the conclusions of this article will be made available by the authors, without undue reservation.

## Ethics Statement

This study was approved by the Ethics Committee of Army Medical Center of PLA affiliated with Army Medical University (Third Military Medical University). The patients/participants provided their written informed consent to participate in this study. Written informed consent was obtained from the individual(s) for the publication of any potentially identifiable images or data included in this article.

## Author Contributions

YW, YN, and QC conceptualized the study and contributed to supervision. YW, YN, GR, and JQ contributed to methodology. GR, JQ, ZX, LC, SY, LL, FXu, GL, MC, YT, MH, LQ, and ZhuZ contributed to formal analysis and investigation. YW, YN, GR, JQ, and YC wrote the original draft. YW, YN, QC, GR, JQ, and YC contributed to writing, reviewing, and editing. YW and YN contributed to funding acquisition. RL, BZ, MZ, JC, FXi, XS, LF, QL, NL, ZQ, and ZhoZ contributed to resources. All authors read and approved the final manuscript.

## Funding

This study was supported by the Key Project of Chongqing Social Livelihood (cstc2018jscx-mszdX0026), the Clinical Technology Innovation Cultivation Program of Army Medical University (Third Military Medical University) (CX2019JS222), the Undergraduate Scientific Research Training Program of Army Medical University (Third Military Medical University) (2020XBK31), and the Key Projects of Chongqing Natural Science Foundation (cstc2020jcyj-zdxmX0025).

## Conflict of Interest

The authors declare that the research was conducted in the absence of any commercial or financial relationships that could be construed as a potential conflict of interest.

## Publisher's Note

All claims expressed in this article are solely those of the authors and do not necessarily represent those of their affiliated organizations, or those of the publisher, the editors and the reviewers. Any product that may be evaluated in this article, or claim that may be made by its manufacturer, is not guaranteed or endorsed by the publisher.

## Supplementary Material

Supplementary Figure 1 The network structure of ResNet50. Resnet50 is composed of four layers, the four layers have 3, 4, 6, and 3 basic blocks, respectively, the first block of each layer is the Downsampling block, and the rest are residual blocks. The internal structure of both blocks is shown on the right. Conv, convolution layer; BN, BatchNorm layer; Relu, the activation function.

Supplementary Figure 2 Comparison of confusion matrix between ResNet50 and endoscopists (per patient and per lesion). Confusion matrix in for deep model, trainee endoscopist (highest accuracy), and competent endoscopist (highest accuracy) at patient and lesion level. **(A,D)** Deep model, **(B,E)** trainee endoscopist, and **(C,F)** competent endoscopist.

Supplementary Figure 3 Heat maps of UC and CD generated by the deep model. Representative endoscopic images of UC **(A,B)** and CD **(C,D)**, including original endoscopic images and CNN processing images.

Supplementary Figure 4 Confusion matrix and ROC curves for multicenter validation (per lesion). Confusion matrix **(A–C)** and ROC curves **(D–F)** for three hospitals. AUC, the area under the receiver operating characteristic curve; ROC, receiver operating characteristic. **(A,D)** The First Affiliated Hospital of Chongqing Medical University, **(B,E)** the Sixth Affiliated Hospital of Sun Yat-sen University, **(C,F)** the Tongji Hospital Affiliated with Huazhong University of Science and Technology.

Supplementary Figure 5 Colonoscopy diagnosis system version 1.0. A website has been made available to provide free access to the deep learning colonoscopy diagnosis system (http://202.202.232.210/ai/default.aspx).

Supplementary Table 1 Results of Six-fold cross-validation on the training dataset (Repeat five times).

Supplementary Table 2 Results of multicentre verification achieved by deep model.

## References

[1] Magro F Gionchetti P Eliakim R Ardizzone S Armuzzi A Barreiro-de Acosta M. Third European Evidence-based Consensus on Diagnosis and Management of Ulcerative Colitis. Part 1: Definitions, Diagnosis, Extra-intestinal Manifestations, Pregnancy, Cancer Surveillance, Surgery, and Ileo-anal Pouch Disorders. J Crohns Colitis. (2017) 11:649-670. 10.1093/ecco-jcc/jjx008 28158501

[2] Gomollon F Dignass A Annese V Tilg H Van Assche G Lindsay JO. 3rd European Evidence-based Consensus on the Diagnosis and Management of Crohn's Disease 2016: Part 1: Diagnosis and Medical Management. J Crohns Colitis. (2017) 11:3-25. 10.1093/ecco-jcc/jjw168 27660341

[3] Flynn S Eisenstein S. Inflammatory bowel disease presentation and diagnosis. Surg Clin North Am. (2019) 99:1051–62. 10.1016/j.suc.2019.08.001 31676047

[4] Su HJ Chiu YT Chiu CT Lin YC Wang CY Hsieh JY Wei SC. Inflammatory bowel disease and its treatment in 2018: global and Taiwanese status updates. J Formos Med Assoc. (2019) 118:1083–92. 10.1016/j.jfma.2018.07.005 30054112

[5] Roushan N Ebrahimi Daryani N Azizi Z Pournaghshband H Niksirat A. Differentiation of Crohn's disease and ulcerative colitis using intestinal wall thickness of the colon: a diagnostic accuracy study of endoscopic ultrasonography. Med J Islam Repub Iran. (2019) 33:57. 10.47176/mjiri.33.57 31456981 PMC6708083

[6] Maaser C Sturm A Vavricka SR Kucharzik T Fiorino G Annese V. ECCO-ESGAR Guideline for Diagnostic Assessment in IBD Part 1: Initial diagnosis, monitoring of known IBD, detection of complications. J Crohns Colitis. (2019) 13:144–64. 10.1093/ecco-jcc/jjy113 30137275

[7] Guo M Wang H Xu S Zhuang Y An J Su C. Alteration in gut microbiota is associated with dysregulation of cytokines and glucocorticoid therapy in systemic lupus erythematosus. Gut Microbes. (2020) 11:1758–73. 10.1080/19490976.2020.1768644 32507008 PMC7524333

[8] Ng SC Chan FK. Infections and inflammatory bowel disease: challenges in Asia. J Dig Dis. (2013) 14:567–73. 10.1111/1751-2980.12091 23875824

[9] Papadakis KA Tabibzadeh S. Diagnosis and misdiagnosis of inflammatory bowel disease. Gastrointest Endosc Clin N Am. (2002) 12:433–49. 10.1016/S1052-5157(02)00005-3 12486937

[10] Mosli M Al Beshir M Al-Judaibi B Al-Ameel T Saleem A Bessissow T. Advances in the diagnosis and management of inflammatory bowel disease: challenges and uncertainties. Saudi J Gastroenterol. (2014) 20:81–101. 10.4103/1319-3767.129473 24705146 PMC3987157

[11] Zheng JJ Cu XQ Shi XH Wang YM Jia LM Zhou XL. Colonoscopic and histologic features of colonic Crohn's disease in Chinese patients. J Dig Dis. (2007) 8:35-41. 10.1111/j.1443-9573.2007.00281.x 17261133

[12] Miehlke S Verhaegh B Tontini GE Madisch A Langner C Münch A. Microscopic colitis: pathophysiology and clinical management. Lancet Gastroenterol Hepatol. (2019) 4:305–14. 10.1016/S2468-1253(19)30048-2 30860066

[13] Jung SA. Differential diagnosis of inflammatory bowel disease: what is the role of colonoscopy? Clin Endosc. (2012) 45:254–62. 10.5946/ce.2012.45.3.254 22977813 PMC3429747

[14] Spiceland CM Lodhia N. Endoscopy in inflammatory bowel disease: Role in diagnosis, management, and treatment. World J Gastroenterol. (2018) 24:4014–20. 10.3748/wjg.v24.i35.4014 30254405 PMC6148432

[15] Gottlieb K Requa J Karnes W Chandra Gudivada R Shen J Rael E. Central reading of ulcerative colitis clinical trial videos using neural networks. Gastroenterology. (2021) 160:710–9 e2. 10.1053/j.gastro.2020.10.024 33098883

[16] Shichijo S Nomura S Aoyama K Nishikawa Y Miura M Shinagawa T. Application of convolutional neural networks in the diagnosis of helicobacter pylori infection based on endoscopic images. EBioMedicine. (2017) 25, 106–11. 10.1016/j.ebiom.2017.10.014 29056541 PMC5704071

[17] Karadag P Morris B Woolfall K. The information and support needs of patients living with inflammatory bowel disease: a qualitative study. Chronic Illn. (2020) 1742395320968617. 10.1177/1742395320968617 33106026 PMC9163778

[18] He J Baxter SL Xu J Xu J Zhou X Zhang K. The practical implementation of artificial intelligence technologies in medicine. Nat Med. (2019) 25:30–6. 10.1038/s41591-018-0307-0 30617336 PMC6995276

[19] Wang P Berzin TM Glissen Brown JR Bharadwaj S Becq A Xiao X. Real-time automatic detection system increases colonoscopic polyp and adenoma detection rates: a prospective randomised controlled study. Gut. (2019) 68:1813–9. 10.1136/gutjnl-2018-317500 30814121 PMC6839720

[20] Reichling C Taieb J Derangere V Klopfenstein Q Le Malicot K Gornet JM. Artificial intelligence-guided tissue analysis combined with immune infiltrate assessment predicts stage III colon cancer outcomes in PETACC08 study. Gut. (2020) 69:681–90. 10.1136/gutjnl-2019-319292 31780575 PMC7063404

[21] Luo H Xu G Li C He L Luo L Wang Z. Real-time artificial intelligence for detection of upper gastrointestinal cancer by endoscopy: a multicentre, case-control, diagnostic study. Lancet Oncol. (2019) 20:1645–54. 10.1016/S1470-2045(19)30637-0 31591062

[22] Tong Y Lu K Yang Y Li J Lin Y Wu D. Can natural language processing help differentiate inflammatory intestinal diseases in China? Models applying random forest and convolutional neural network approaches. BMC Med Inform Decis Mak. (2020) 20:248. 10.1186/s12911-020-01277-w 32993636 PMC7526202

[23] Kim JM Kang JG Kim S Cheon JH. Deep-learning system for real-time differentiation between Crohn's disease, intestinal Behcet's disease, and intestinal tuberculosis. J Gastroenterol Hepatol. (2021) 36:2141–8. 10.1111/jgh.15433 33554375

[24] Ding Z Shi H Zhang H Meng L Fan M Han C. Gastroenterologist-level identification of small-bowel diseases and normal variants by capsule endoscopy using a deep-learning model. Gastroenterology. (2019) 157:1044–54 e5. 10.1053/j.gastro.2019.06.025 31251929

[25] Stidham RW Liu W Bishu S Rice MD Higgins PDR Zhu J. Performance of a deep learning model vs. human reviewers in grading endoscopic disease severity of patients with ulcerative colitis. JAMA Netw Open. (2019) 2:e193963. 10.1001/jamanetworkopen.2019.3963 31099869 PMC6537821

[26] Tang A Tam R Cadrin-Chenevert A Guest W Chong J Barfett J. Canadian association of radiologists white paper on artificial intelligence in radiology. Can Assoc Radiol J. 69:120–35 (2018). 10.1016/j.carj.2018.02.002 29655580

[27] Calderaro J Kather JN. Artificial intelligence-based pathology for gastrointestinal and hepatobiliary cancers. Gut. (2021) 70:1183–93. 10.1136/gutjnl-2020-322880 33214163

