# Supplementary figures and images for: Development and Validation of a Deep Neural Network for Accurate Identification of Endoscopic Images From Patients With Ulcerative Colitis and Crohn's Disease
Source: Front Med (Lausanne). 2022 Mar 18;9:854677. doi: 10.3389/fmed.2022.854677 (PMC8974241; doi:10.3389/fmed.2022.854677)

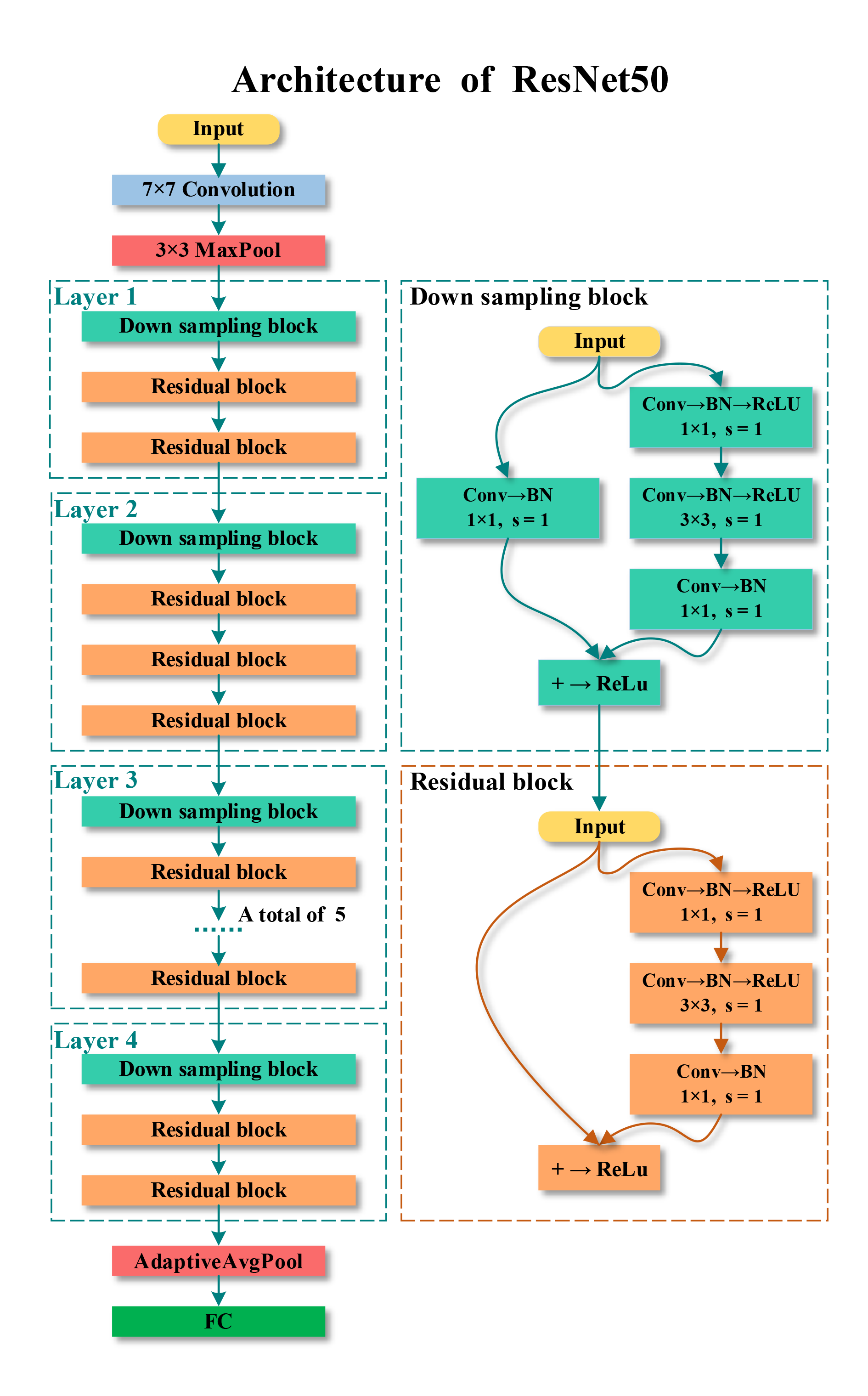

Supplement: Supplementary Figure 1 — The network structure of ResNet50. Resnet50 is composed of four layers, the four layers have 3, 4, 6, and 3 basic blocks, respectively, the first block of each layer is the Downsampling block, and the rest are residual blocks. The internal structure of both blocks is shown on the right. Conv, convolution layer; BN, BatchNorm layer; Relu, the activation function. [file Image_1.TIF]

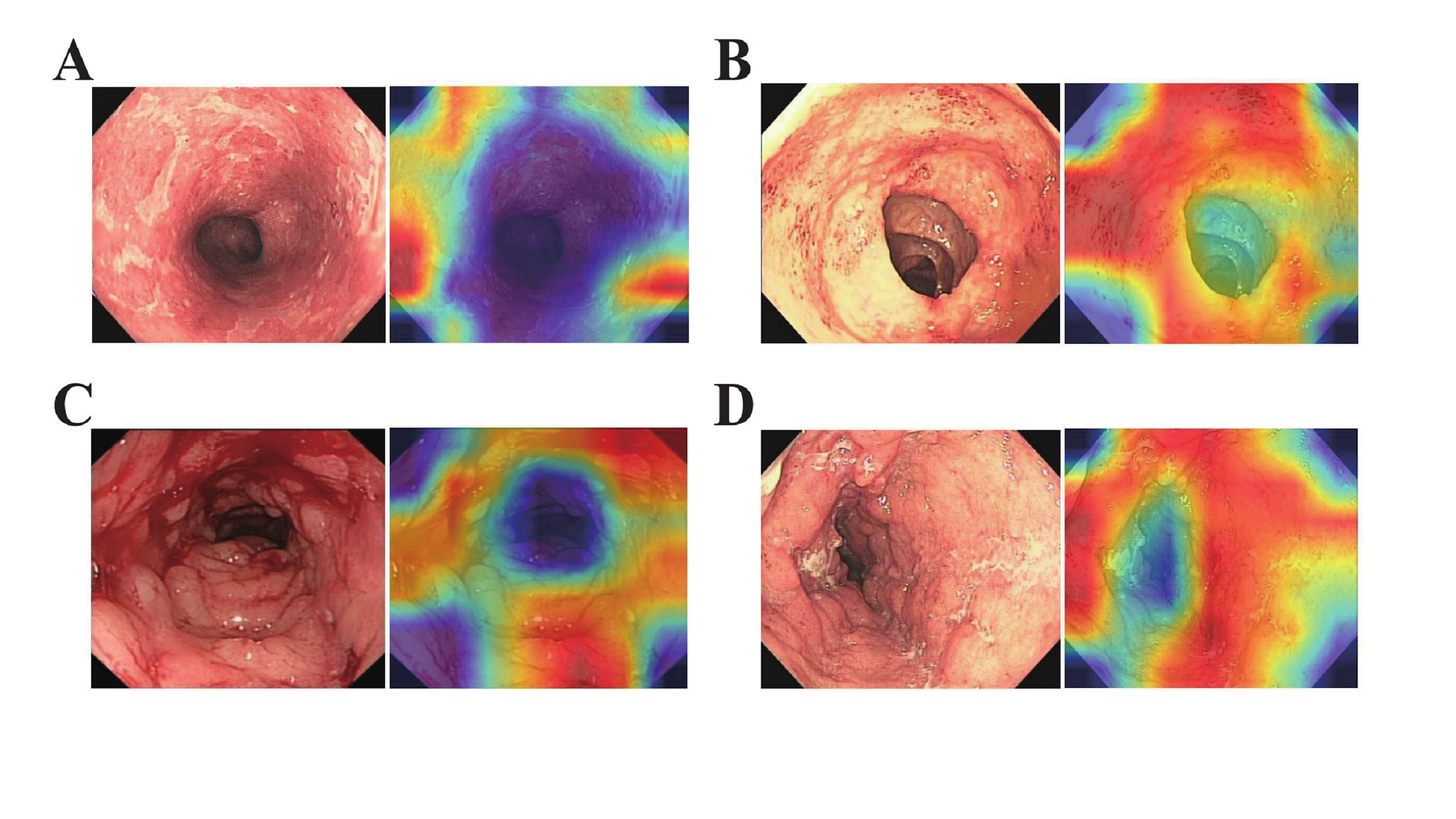

Supplement: Supplementary Figure 3 — Heat maps of UC and CD generated by the deep model. Representative endoscopic images of UC (A,B) and CD (C,D), including original endoscopic images and CNN processing images. [file Image_3.TIF]

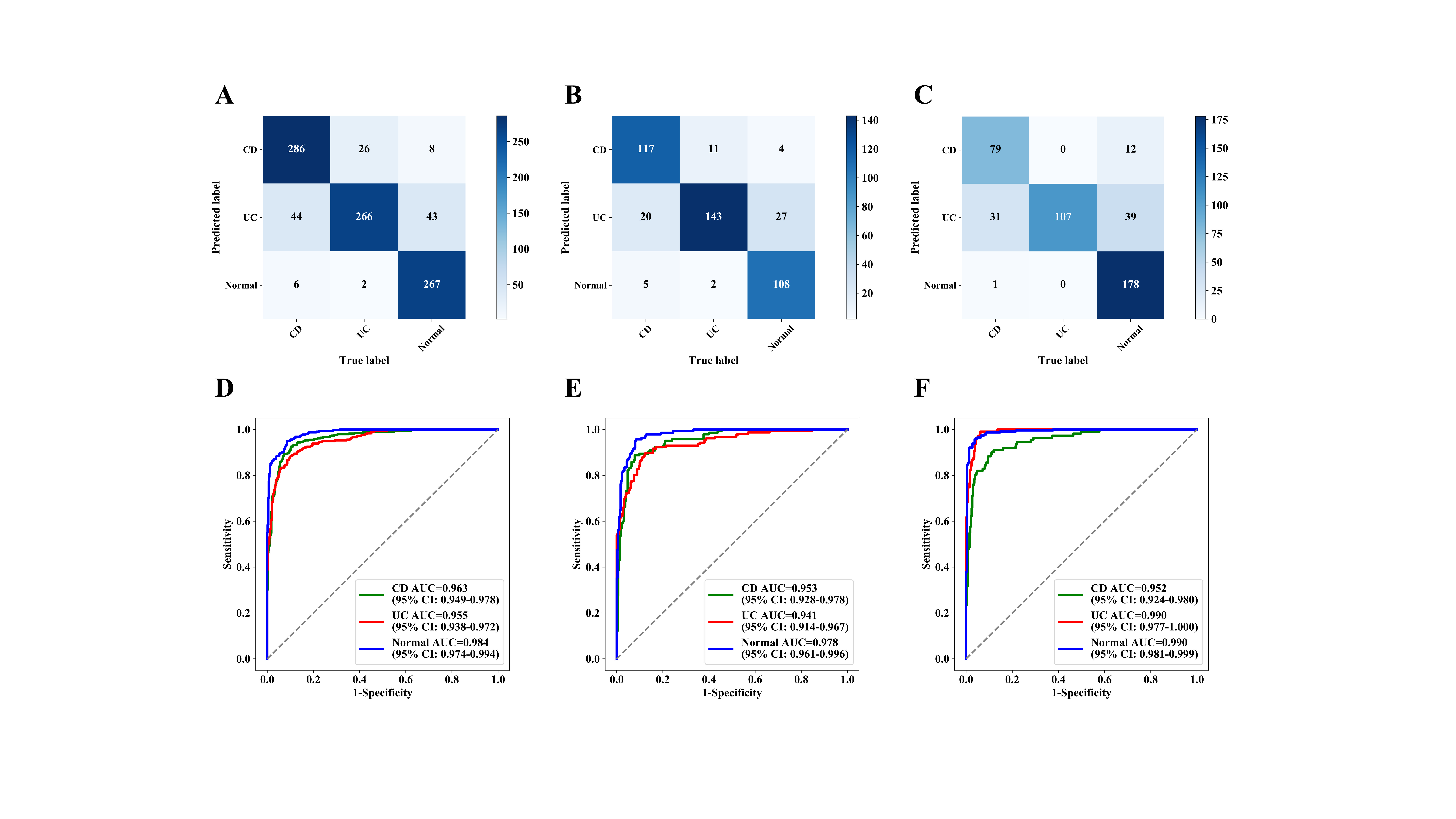

Supplement: Supplementary Figure 4 — Confusion matrix and ROC curves for multicenter validation (per lesion). Confusion matrix (A–C) and ROC curves (D–F) for three hospitals. AUC, the area under the receiver operating characteristic curve; ROC, receiver operating characteristic. (A,D) The First Affiliated Hospital of Chongqing Medical University, (B,E) the Sixth Affiliated Hospital of Sun Yat-sen University, (C,F) the Tongji Hospital Affiliated with Huazhong University of Science and Technology. [file Image_4.TIF]

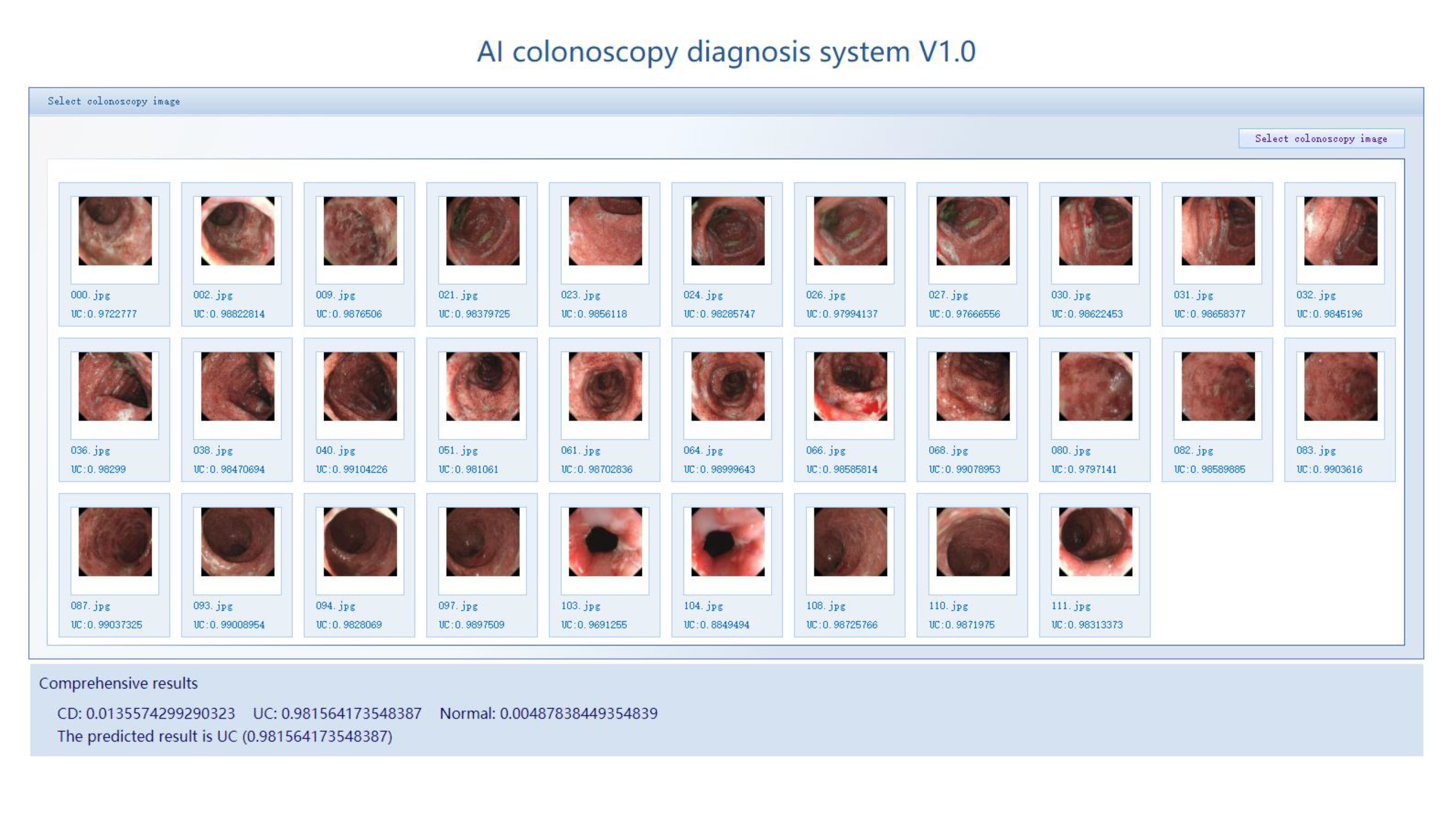

Supplement: Supplementary Figure 5 — Colonoscopy diagnosis system version 1.0. A website has been made available to provide free access to the deep learning colonoscopy diagnosis system (http://202.202.232.210/ai/default.aspx). [file Image_5.TIF]
