# Supplementary material for: Development and Validation of a Deep Neural Network for Accurate Identification of Endoscopic Images From Patients With Ulcerative Colitis and Crohn's Disease
Source: Front Med (Lausanne). 2022 Mar 18;9:854677. doi: 10.3389/fmed.2022.854677 (PMC8974241; doi:10.3389/fmed.2022.854677)
Supplement: Supplementary Table 1 — Results of Six-fold cross-validation on the training dataset (Repeat five times). [file Data_Sheet_1.pdf]

Supplemental Tables

Supplement Table 1 Results of Six-fold cross-validation on the training dataset (Repeat five times)

	Accuracy(95%CI)		Sensitivity(95%CI)	Specificity(95%CI)	PPV (95%CI)	NPV (95%CI)	AUC (95%CI)
**Per patient**	0.962(0.951-0.971)	**Normal**	0.989(0.969-0.996)	0.999(0.995-1.000)	0.996(0.980-0.999)	0.997(0.992-0.999)	1.000(0.998-1.000)
		**UC**	0.959(0.940-0.972)	0.970(0.956-0.980)	0.961(0.942-0.974)	0.969(0.954-0.979)	0.988(0.982-0.995)
		**CD**	0.972(0.953-0.983)	0.964(0.950-0.975)	0.939(0.915-0.957)	0.984(0.973-0.990)	0.988(0.981-0.995)
**Per lesion**	0.916(0.913-0.920)	**Normal**	0.985(0.983-0.987)	0.987(0.985-0.989)	0.978(0.975-0.981)	0.991(0.990-0.993)	0.999(0.999-0.999)
		**UC**	0.903(0.897-0.909)	0.922(0.918-0.925)	0.842(0.835-0.849)	0.954(0.951-0.957)	0.974(0.971-0.976)
		**CD**	0.935(0.929-0.939)	0.912(0.908-0.916)	0.832(0.825-0.839)	0.968(0.965-0.970)	0.977(0.975-0.980)

PPV, positive predictive value; NPV, negative predictive value; AUC, area under the receiver operating characteristic curve.

Supplement Table 2 Results of multicentre verification achieved by deep model

Hospital		Accuracy(95%CI)		Sensitivity(95%CI)	Specificity(95%CI)	PPV (95%CI)	NPV (95%CI)
The First Affiliated Hospital of Chongqing Medical University	Per lesion	0.864(0.841-0.884)	Normal	0.950(0.920-0.969)	0.914(0.890-0.934)	0.848(0.807-0.882)	0.973(0.957-0.983)
			UC	0.884(0.843-0.916)	0.899(0.874-0.920)	0.798(0.751-0.838)	0.945(0.925-0.961)
			CD	0.926(0.892-0.949)	0.897(0.870-0.919)	0.832(0.790-0.866)	0.956(0.936-0.970)
	Per patient	0.951(0.880-0.981)	Normal	1.000(0.875-1.000)	1.000(0.934-1.000)	1.000(0.875-1.000)	1.000(0.934-1.000)
			UC	0.963(0.817-0.993)	0.963(0.875-0.990)	0.929(0.774-0.980)	0.981(0.901-0.997)
			CD	0.926(0.766-0.979)	0.981(0.902-0.997)	0.962(0.811-0.993)	0.964(0.877-0.990)
The Sixth Affiliated Hospital of Sun Yat-sen University	Per lesion	0.842(0.805-0.873)	Normal	0.957(0.909-0.980)	0.916(0.879-0.943)	0.842(0.777-0.890)	0.978(0.954-0.990)
			UC	0.897(0.840-0.936)	0.875(0.832-0.909)	0.800(0.735-0.853)	0.939(0.903-0.962)
			CD	0.887(0.825-0.929)	0.922(0.886-0.947)	0.846(0.779-0.895)	0.944(0.912-0.966)
	Per patient	0.942(0.871-0.975)	Normal	1.000(0.871-1.000)	0.967(0.886-0.991)	0.929(0.774-0.980)	1.000(0.938-1.000)
			UC	0.967(0.833-0.994)	0.964(0.879-0.990)	0.935(0.793-0.982)	0.982(0.904-0.997)
			CD	0.967(0.833-0.994)	0.964(0.879-0.990)	0.935(0.793-0.982)	0.982(0.904-0.997)
Tongji Hospital	Per lesion	0.814(0.776-0.848)	Normal	0.956(0.921-0.976)	0.963(0.929-0.981)	0.965(0.932-0.982)	0.955(0.918-0.975)
			UC	0.991(0.949-0.998)	0.938(0.907-0.959)	0.835(0.760-0.889)	0.997(0.983-0.999)
			CD	0.910(0.842-0.950)	0.881(0.842-0.911)	0.716(0.637-0.784)	0.967(0.941-0.982)
	Per patient	0.897(0.736-0.964)	Normal	1.000(0.701-1.000)	0.950(0.764-0.991)	0.900(0.596-0.982)	1.000(0.832-1.000)
			UC	1.000(0.723-1.000)	1.000(0.832-1.000)	1.000(0.723-1.000)	1.000(0.832-1.000)
			CD	1.000(0.723-1.000)	0.947(0.754-0.991)	0.909(0.623-0.984)	1.000(0.824-1.000)

PPV, positive predictive value; NPV, negative predictive value.
